# Developmental Analysis of Prospective Effects of Problem Drinking and Health Problems in Three-Age Groups

**DOI:** 10.1093/geroni/igab046.3586

**Published:** 2021-12-17

**Authors:** Thomas Kwan, Douglas Bowlby, Matthew Lee

**Affiliations:** 1 Rutgers University, Manalapan, New Jersey, United States; 2 Rutgers University, Rutgers University, New Jersey, United States; 3 Rutgers University, Piscataway, New Jersey, United States

## Abstract

Past research has clearly demonstrated interrelations between drinking and health. However, little research has investigated this from a lifespan-development perspective, which is the objective of the current study. Our hypotheses predicted results consistent with the familiar “J-shaped curve” of drinking effects on health, including that health problems would be (1) lower in moderate drinkers than abstainers and (2) higher in excessive drinkers than moderate drinkers. We also hypothesized that these protective effects of moderate drinking would increase with age across the lifespan. The current study used two waves of data from a large U.S.-representative sample. Analyses used 3*3 between-persons ANCOVAs that tested a three-level Wave-1 drinking-group factor and a three-level Wave-1 age-group factor. Of particular importance were the drinking-group-by-age interactions. Various Wave-2 health outcomes were predicted in different ANCOVAs, and each ANCOVA controlled for Wave-1 levels of the Wave-2 health outcome. Across nearly all health outcomes, young adults did not show significant differences between abstainers and moderate drinkers, whereas midlife and older adults consistently showed better health for moderate drinking versus abstainers. This suggests that protective effects of moderate drinking apply more-so to midlife and older adults than young adults. Surprisingly, excessive drinkers generally did not show poorer health than moderate drinkers, except for mixed evidence for such effects only among older adults. Thus, only older adults showed patterns entirely consistent with our hypothesized “J-shaped curve.” A next analytic step we will conduct in advance of this poster presentation will assess if alternative excessive-drinking operationalizations more consistently signal health problems.

